# Society Meetings and Transactions for 1892

**Published:** 1893-01

**Authors:** 


BUFFALO MEDICAL AND SURGICAL JOURNAL
A MONTHLY REVIEW OF MEDICINE AND SURGERY.

*EDITORS:*
THOMAS LOTHROP, M. D. - - WM. WARREN POTTER, M. D.
All communications, whether of a literary or business character, should be addressed to the managing editor: 284 FRANKLIN STREET, BUFFALO, N. Y.

VOL. XXXII. JANUARY, 1893. No. 6.

### SOCIETY MEETINGS AND TRANSACTIONS FOR 1892.

As THE years advance, there is a constantly increasing improvement in medical society work, and in the publication of the transactions of the several medical societies that issue annual volumes. This applies to local, State, and National organizations almost without exception, and is particularly instanced in the excellent work done in the year 1892. The several volumes of transactions that have already appeared give abundant evidence of the quality as well as quantity thereof, and we have no doubt that those yet to appear will fully maintain, if not excel, the standards of the same societies for previous years. This is as it should be, for in reality these society meetings, especially those of the State and National organizations, are post-graduate courses to which general practitioners as well as specialists may go and learn. There is a constant increase in the attendance of these societies, and we regard this fact as one of the most healthful signs of a tendency toward a higher order of medical education. Many physicians cannot spare the time to be absent from six to twelve weeks during the year to attend one of the great metropolitan post-graduate schools, but all may and should spare three days now and three days again in attending two or three society meetings during the year. To attend such meetings will serve to keep them in touch with the progress of medicine, and to burnish up such rusty links in the chain of professional brotherhood as need repolishing.

Most of the National special societies issue general invitations to the profession at large to attend their meetings, and we regard it important for every physician who resides within a reasonable distance from the place appointed for any such meeting, to attend it as regularly and as studiously as though he were a member. The object of these meetings is not alone for the benefit of the men who belong to the societies, but to improve the whole profession, as far as they may be able to contribute to that desirable end. There is no more valuable literature than the annual transactions of most of the special societies and some of the State organizations. Too little attention is paid to the purchase of these volumes by the profession, and too much money is expended on books that do not contain anything like the amount of valuable information. It is all very well for some authors of papers to make haste to publish them in the weekly or monthly magazines soon after reading them in one of these societies, but, as a matter of fact, the experienced writer and society debater relies on the annual volumes of transactions of the societies in which he is interested, where all the papers and discussions are grouped together, and are readily and conveniently accessible whenever he wishes to refer to them. The fact is, that medical journals are rendering their columns less and less interesting by the publication of syndicate papers that are read at societies, for one soon gets tired of seeing Dr. Blank's article published in eight or ten journals coming to his table during a single month.

But, we are now getting beyond the purpose that furnished the inspiration of this article, and will reserve for another time what we may have to say on the journalistic side of the question. For the present, we are content to call attention to the good work, for the most part, done by American societies, National, State, and local, for the year 1892, and to bespeak from the young men of the profession a careful examination of the literature that has emanated from these several bodies. Furthermore, we predict that they may look forward to 1893 for even further and greater gain in respect to quality of the society work done, and of the admirable way in which it will be published in the several transactions. When a society does not properly report and publish its work, its chief influence falls to the ground. No society can maintain a proper *esprit de corps* that does not furnish *verbatim* reports of its proceedings, which must include the papers read and the discussions that follow them. Most of the National societies do this work admirably, and some of the State societies are following closely their example. Only a few of the local societies, however, have thus far succeeded in accomplishing near what ought to be done in this direction, and of these we may name the New York Academy of Medicine and the Philadelphia County Medical Society as models. The papers read and discussions held in these societies are reported in full and furnished the periodicals for publication, in a properly edited manner, making them available to the professional world. They are then preserved in book form by the societies for future reference. This involves the employment of a careful secretary and providing compensation for him, *but it pays.*

